# PRRSV detection by qPCR in processing fluids and serum samples collected in a positive stable breeding herd following mass vaccination of sows with a modified live vaccine

**DOI:** 10.1186/s40813-020-00186-8

**Published:** 2021-01-04

**Authors:** A. Lebret, P. Berton, V. Normand, I. Messager, N. Robert, F. Bouchet, M. Brissonnier, G. Boulbria

**Affiliations:** 1 Porc. Spective Swine Vet Practice, ZA de Gohélève, 56920 Noyal-Pontivy, France; 2 rezoolution Pig Consulting Services, ZA de Gohélève, 56920 Noyal-Pontivy, France; 3 Boehringer Ingelheim Animal Health France, Swine Bussiness Unit, 16, rue Louis Pasteur, 44119 Treillères, France

**Keywords:** Swine, PRRSV, Processing fluid, Modified-live vaccination, Monitoring

## Abstract

In the last two decades, in France, Porcine Reproductive and Respiratory Syndrome Virus (PRRSV) stabilization protocols have been implemented using mass vaccination with a modified live vaccine (MLV), herd closure and biosecurity measures. Efficient surveillance for PRRSV is essential for generating evidence of absence of viral replication and transmission in pigs. The use of processing fluid (PF) was first described in 2018 in the United States and was demonstrated to provide a higher herd-level sensitivity compared with blood samples (BS) for PRRSV monitoring. In the meantime, data on vertical transmission of MLV viruses are rare even as it is a major concern. Therefore, veterinarians usually wait for several weeks after a sow mass vaccination before starting a stability monitoring. This clinical study was conducted in a PRRSV-stable commercial 1000-sow breed-to-wean farm. This farm suffered from a PRRS outbreak in January 2018. After implementing a stabilisation protocol, this farm was controlled as stable for more than 9 months before the beginning of the study. PF and BS at weaning were collected in four consecutive batches born after a booster sow mass MLV vaccination. We failed to detect PRRSV by qPCR on PF and BS collected in a positive-stable breeding herd after vaccination with ReproCyc® PRRS EU (Boehringer Ingelheim, Ingelheim, Germany).

## Background

Porcine Reproductive and Respiratory Syndrome (PRRS) has a significant impact on the health and welfare of pigs and has become enzootic in most pig production areas [18]. Improvements in detection and management of PRRS virus (PRRSV) in production systems continue to be challenging for swine producers and veterinarians.

In the last two decades, in France, stabilization protocols have been developed using mass vaccination with a modified live vaccine (MLV), herd closure and biosecurity measures strengthening [1].

To assess the success of such protocols, the first step is to evaluate the status of the breeding herd, usually classified as naive, stable or unstable. This classification is described by the American Association of Swine Veterinarians (AASV) guidelines [4] and is based on blood samples (BS) collected from due-to-wean piglets and tested by qPCR pooled by five. This method aims to generate evidence of absence of viral replication and transmission in pigs.

Recent field studies in commercial farms suggested that qPCR on 30 blood samples collected from piglets prior to weaning lack sensitivity to detect PRRSV in low prevalence farms [5, 7, 8, 15]. Other biological samples such as oral fluids [5, 7], udder wipes [17], umbilical cords [12], and processing fluids (PF) [9, 11, 15, 16] have been assessed. Sampling more animals resulted in an increase of the herd-level sensitivity and a reduction of the diagnostic cost. A scheme for considering a herd as stable has been suggested using PF, consisting in testing PF for PRRSV RNA by qPCR for at least eight negative consecutive weeks followed by a qPCR test in BS in due-to-wean piglets [15].

Naïve sows’ PRRSV infections during the third gestation trimester lead to the birth of viraemic piglets, which contribute to the dissemination of the virus during the suckling period [2]. Moreover, pharmaceutical companies reported in the summaries of product characteristics that vaccine strains from PRRS MLV vaccines are detected in newborn piglets when vaccinating naive gilts during last third of gestation. Therefore when implementing a sow mass vaccination (SMV), veterinarians usually wait for several weeks before to start a stability monitoring. This period may vary between 9 weeks [15] and 12 weeks [1] after a sow mass vaccination with a PRRS MLV vaccine.

The aim of this clinical study was to describe vaccinal strain detection using qPCR on PF and serum samples collected from piglets born within the 2 months following a sow mass vaccination with ReproCyc® PRRS EU (Boehringer Ingelheim, Ingelheim, Germany) in a positive stable breeding herd.

## Materials and methods

### Study design

This study was conducted in a commercial 1000-sow breed-to-wean farm located in Brittany (France) implementing a 2-week batch farrowing system. After a PRRSV-1 outbreak in January 2018, a stabilization protocol including MLV vaccination and herd closure was implemented in February 2018. First vaccination concerned all gilts, boars and sows present in the breeding unit simultaneously. This vaccination scheme was repeated 3 weeks later as previously described [1]. Then, booster MLV vaccinations were implemented every 16 weeks. In total, 4 boosters SMV were performed after the first vaccination before the beginning of our clinical study. In this farm, all sow mass vaccinations (including due-to-farrow sows) were performed using ReproCyc® PRRS EU (2 mL, intra-muscular route - Boehringer Ingelheim, Ingelheim, Germany).

**Fig. 1:**
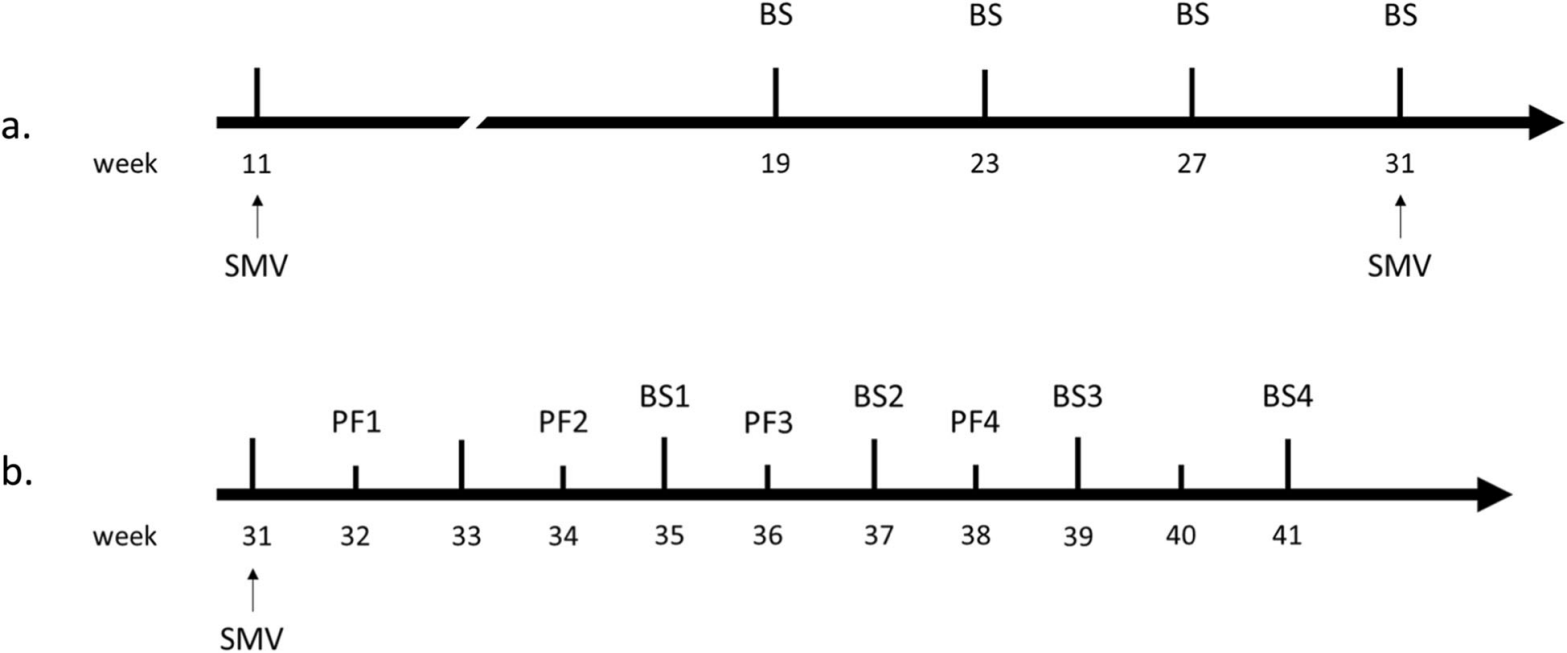
Timeline of the case report in 2019. **a**. Pre-study stability monitoring. **b**. Samples collection after SMV (sow mass vaccination) [PFx=processing fluids collected from batch x; BSx=blood sample collected from batch x]

According to the AASV classification [4], the farm was controlled positive stable before the study. After a sow mass vaccination in the 11th week of 2019 (week 11), blood samples from 30 due-to-wean piglets were tested negative by qPCR on four batches (on week 19, 23, 27 and 31) over a 90-day period (Fig. 1a). On the same time, no clinical sign consistent with PRRS was observed in the breeding herd (including gilts, sows and sucklers).

Then, a SMV was implemented the week before this study started, on week 31 of 2019, with ReproCyc® PRRS EU, using one sterile needle per sow. Sampling started the week following sow vaccination and continued every 2 weeks until processing fluids and blood samples tested negative for PRRSV-1 on four consecutive batches (Fig. 1b).

### Sample collection

Within each batch, processing fluids, described as the serosanguinous fluid recovered at the time of castration and tail docking [9], were collected in plastic bags from three-to-five-day-old piglets. Each bag contained testicles and tails from all the piglets from a single litter. At the end of processing, fluids were stored in a 50 mL plastic tube and frozen in the farm (− 18 °C).

The day prior to weaning, blood samples were collected from a convenience sample of 30 piglets, targeting the weakest piglet within the litter (one piglet per litter). Blood samples were collected in plain test tubes using one sterile needle per piglet from the cranial vena.

All frozen processing fluids (*n* = 75 per batch) and blood samples (*n* = 30 per batch) were submitted to the laboratory the day of blood sampling within 2 h after collection.

### Diagnostic testing

Diagnostic testing was performed at Labofarm (Finalab Veterinary Laboratories Group, Loudéac, France). Blood samples were centrifuged to separate serum (4500 g for 5 min). For processing fluids, samples were treated using assays routinely used for swine oral fluids [9]. All samples were tested for PRRSV RNA using Adiavet PRRSV real time 100R kit (BioX Diagnostics, Rochefort, Belgium). Processing fluids were pooled by 15 litters (approximately 220–250 piglets processed) and serum samples were pooled by five litters (one serum per litter). A sample was considered positive if the cycle threshold (Ct) value was ≤40 for serum and processing fluid.

## Results

A total of 300 processing fluids and 120 serum samples, corresponding to the monitoring of the first 4 production batches following the sow mass vaccination, were collected. On average, 14.7 (SD ±0.9) piglets per litter were included in the collection of PF. Finally, 20 pools of processing fluids and 24 pools of blood samples were analysed. All pooled samples submitted for qPCR test were negative.

## Discussion

This clinical study was performed in only one PRRSV positive stable sow herd in a farrowing unit with a high biosecurity level, after a mass vaccination with a specific MLV sow vaccine. The study was designed in order to investigate MLV virus circulation using conventional sampling procedure used in the field by practitioners.

During the study period, no PRRSV was detected in processing fluids aggregated from 75 litters and collected one, three, five and 7 weeks after mass vaccination of the sows. Even if we could expect a vertical transmission from sow to piglets, no PRRSV RNA was detected in all these samples. No previous report supported the fact that vertical transmission was possible in non naïve sows after a MLV vaccination but in practice, postponing the stability monitoring after a SMV is common. In addition, no horizontal transmission from sow to piglets and within piglets in the batch was observed as no PRRSV RNA was detected in serum samples collected from due-to-wean piglets born after the SMV. However, we did not prove the lack of horizontal transmission to piglets aged 2-week-old on vaccination day. Indeed, our study focused on MLV virus detection testing PF and BS only in the batches born after the SMV. Our results must be considered cautiously because sampling procedure - four testings in thirty due-to-wean piglets pooled by five and 75 PF pooled by 15 out of 90 litters per batch – doesn’t allow to detect low prevalence but this procedure is representative of field conditions.

In our study, PF were pooled by 15 litters which is quite low regarding the common practice [15]. So, the probability that pooling could have affected PCR detection is low as previously described [16]. In experimental conditions, pooling serum samples by five would decrease the sensitivity of PRRSV detection by 6% [13]. However, PCR on pools of five sera is commonly accepted because it didn’t seem to affect PRRSV detection at batch level in field conditions [7].

Processing fluids are useful to detect PRRSV presence at the time of piglet castration and tail docking, three to 5 days after birth [15]. In unstable farms, sampling of newborn piglets is valid to determine whether or not vertical transmission has occurred [12]. Detection of PRRSV RNA in processing fluids, collected from all pigs submitted to processing, using qPCR increase the probability of PRRSV detection [9] compared with 30 blood samples collected from due-to-wean piglets. This is true if the number of piglets’ samples included in the pool did not exceed 352 [10]. In contrast to that, a previous study demonstrated that screening the newborn population was not sufficient to consider a herd stable for PRRSV [15]. In our study, all the pooled samples turned out negative. In the tested piglets’ batches, using sampling procedures commonly used in field condition, we could not evidence transmission of the vaccine strain, neither vertical, nor horizontal. A study conducted in 35 breeding herds in Spain monitored 58 SMV using Unistrain® PRRS (Hipra, Amer, Spain). Considering that Torrents’ protocol study and ours were not strictly comparable, the authors observed occasional positive qPCR results in the following sampling just after a MLV SMV [14]. In this study, in positive stable farms, these positive results were not repeated in further sampling events. Moreover they were unable to sequence the virus because of high Ct. The authors suggested that this could be indicative of low levels of PRRSV vaccine strain circulation in due-to-wean piglets even if they couldn’t prove it. In our study, conducted in only one farm and after a single SMV event using a different vaccine, we could not demonstrate this low circulation in the tested batches born after the SMV. We did not test the suckling piglets born 2 weeks before the SMV as sometimes Torrents did. So, we are not able to conclude and discuss about horizontal transmission during this period.

To our knowledge, data on vertical transmission of MLV virus are rare even if it is a major concern for swine practitioners and producers. Our study suggests that samples could be taken after the vaccination protocol was implemented in positive stable farms. This indicates that stability monitoring of positive stable MLV vaccinated farms could be performed without taking into account the last SMV event.

Moreover, recombination events between different PRRSV-1 vaccine strains were previously reported [3, 6]. Eclercy described that a recombinant was discovered in a farm after successive vaccinations of growing pigs with two different MLVs few weeks apart. To limit the risk of recombination, the absence of vaccine strain viremia in piglets weaned from PRRS MLV vaccinated sows would be preferable before their vaccination when needed. In multisite systems, piglets are often delivered without information about the vaccine used in their dams and can be vaccinated with another MLV strain.

Proper management of vaccination events in sow farms, internal biosecurity and knowledge on vaccine strain circulation are critical points to consider when designing a PRRSV control and monitoring protocol.

## Conclusion

In the condition of our study, we did not detect any PRRSV in piglets born after mass vaccination of the breeding herd with ReproCyc® PRRS EU (Boehringer Ingelheim, Ingelheim, Germany), a specific sow MLV vaccine. Additional investigations should aim to assess the vaccine strain circulation in more herds and with all other MLVs available on the European market. This information would help practitioners in their choice when implementing vaccinal strategies against PRRSV: first, in order to adapt the timeline of herd stability monitoring; secondly, to avoid PRRSV vaccine strain viremia if the weaners have to be vaccinated.

## Data Availability

All datasets used in this study are available from the corresponding author on reasonable request.
